# Assessment of the potential radiation hazards posed by Nubian sandstone, Egypt

**DOI:** 10.1038/s41598-023-47150-4

**Published:** 2023-12-01

**Authors:** Ahmed E. Abdel Gawad, Hassan Eliwa, Masoud S. Masoud, Mayeen Uddin Khandaker, Mohamed Y. Hanfi

**Affiliations:** 1 Nuclear Materials Authority, P.O. Box 530, El-Maadi, Cairo Egypt; 2 Geology Department, Faculty of Science, Minufiya University, Shebin El Kom, Egypt; 3 Centre for Applied Physics and Radiation Technologies, School of Engineering and Technology, Sunway University, 47500 Bandar Sunway, Selangor Malaysia; 4 Faculty of Graduate Studies, Daffodil International University, Daffodil Smart City, Birulia, Savar, Dhaka 1216 Bangladesh; 5 Ural Federal University, St. Mira, 19, Yekaterinburg, Russia 620002

**Keywords:** Environmental sciences, Natural hazards, Risk factors

## Abstract

The study found that the activity concentrations of the radionuclides ^238^U, ^232^Th and ^40^K in the sandstone are 32 ± 13, 29.6 ± 12.2, and 132.6 ± 86.4 Bq kg^−1^, respectively. These values are lower than the reported worldwide limits of 33, 45, and 412 Bq kg^−1^. According to the present study, the absorbed dose rate (D_air_), the annual effective dose, and the excess life time cancer were all found to be below the worldwide mean. Pearson correlation, PCA, and HCA were used to analyze the data and identify patterns in the relationship between radionuclides and radiological hazards. A statistical analysis of the sandstones showed that the radioactive elements ^238^U, ^232^Th and ^40^K are the main contributors to the radioactive risk. The study suggests that the sandstone is safe to use. The levels of radioactivity are not high enough to pose a risk to human health.

## Introduction

The rapid growth of population and the expansion of infrastructure have put a strain on the urban environment, and chemical pollution is one of the most serious problems^1,2^. The sources of radioactivity in the environment can be subdivided into two main categories: natural and artificial^3,4^. Natural sources of radioactivity include rocks, soil, water, and air. These sources contain radioactive elements that have been present on Earth since its formation. Artificial sources of radioactivity include nuclear power plants, medical imaging devices, and nuclear weapons. These sources emit radioactivity as a result of human activities^5–7^. The main sources of radiation in our environment are primordial radionuclides, which are radioactive elements that have been around since the formation of the Earth. These radionuclides have very long half-lives, meaning that they take a very long time to decay^8–10^. The radionuclides are released from the parent rock and soil through a variety of processes, such as weathering, erosion, micro-fractures, joints, faults, shear zones and hydrothermal alteration. The way these processes occur determines how the radionuclides are distributed in space^11,12^. The transmission of radionuclides from rocks and soil to people can occur through inhalation, ingestion, and dermal contact. These pathways can expose people to high doses of radiation, which can increase the risk of cancer and other health problems^13,14^. Background radiation levels can vary depending on the location, so it is important to have this information in order to assess the potential risks of radiation exposure^15,16^. The levels of radioactivity in soil from these three radionuclides are relatively low, with mean values of 33, 45, and 412 Bq kg^−1^, respectively. The exposure rate from gamma radiation from the ground can vary depending on the location, but it is typically around 59 nGy h^−1^^17,18^. The total exposure rate is calculated by taking into account the amount of natural radiation from the ground, cosmic rays, and other sources^19,20^.

Sandstones have varying degrees of durability and colors could be due to cementing and/or binding materials between particles. These rocks have been widely used as a good building material especially in regions of compact rocks. They were used in many glass by low-cost, road construction, concrete products, cement, bricks, natural bio-based construction materials, gothic monuments, schools, villa developments, elegant terrace, tenement fronts, churches, and commercial and public buildings^21–26^. On the other hand, Sandstone rocks contain voids or pores that could play an important role of fluids which are characterized by differing physical properties and permit these fluids to movement. They have ability for carrying fluids such as oil, gas and water, which related to variability in both porosity and permeability^27^. However, Sandstones are considered as one sedimentary rock hosted U-deposits include basal-type, tabular, roll-front and tectono-lithologic deposits^28^.

The Fileita area is a part of a major graben of Wadi Jararah–Wadi Kharit region. It is situated in the southwestern corner of the Eastern Desert, Egypt. The region is of considerable interest to many researchers since the discoveries of many granites bearing rare-metal mineralization^29–36^.

The present work aims are twofold: study of (1) the geological and petrographical features of Fileita Nubian Sandstone (2) the activity concentrations of natural radionuclides ^238^U, ^232^Th and ^40^K in Timsah Formation and Um Baramil Formation at Fileita area.

## Geologic setting

**Figure 1:**
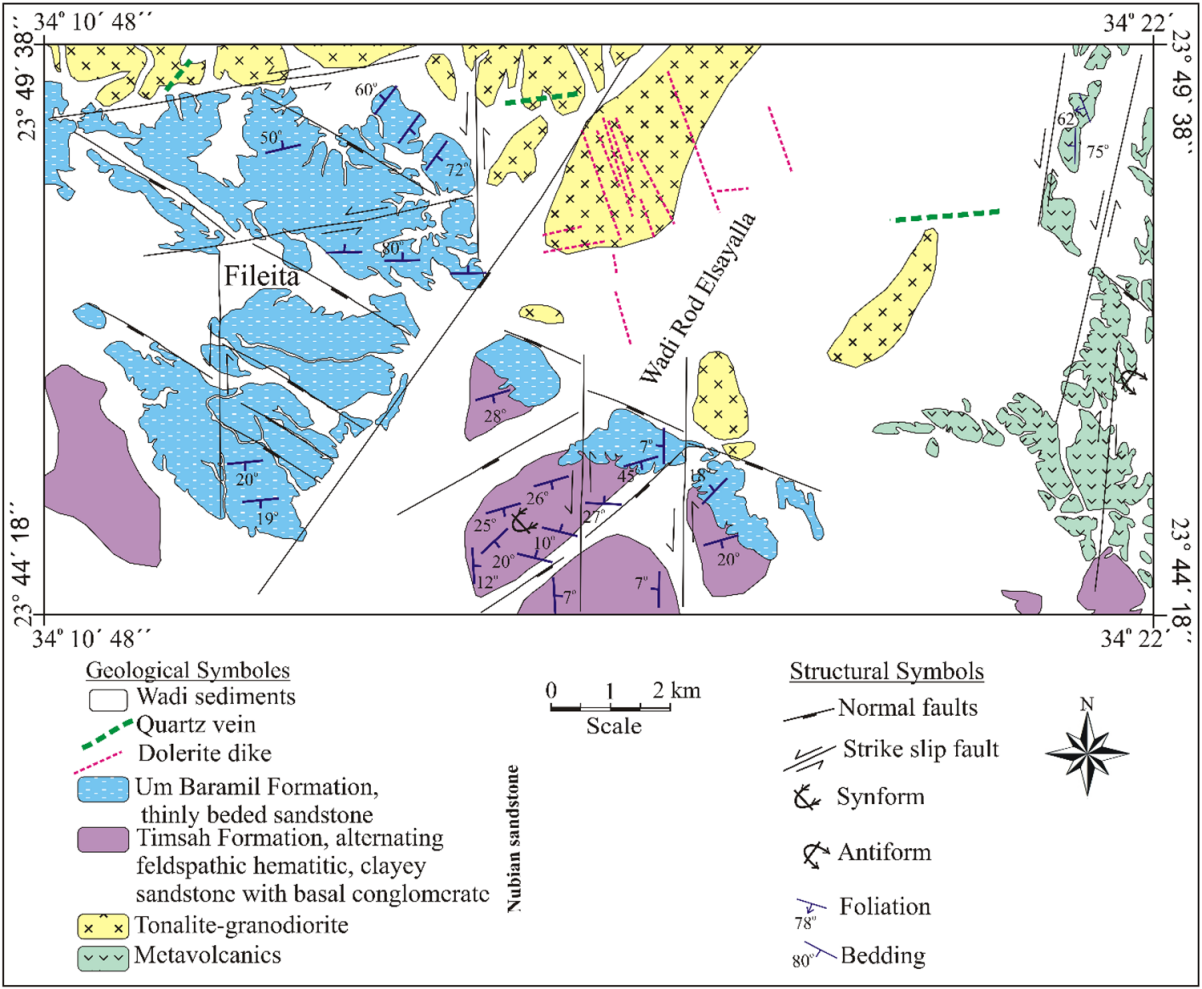
Geologic map of Fileita area, South Eastern Desert of Egypt.

Nubian sandstones comprise two formations include Early Timsah Formation and Late Um Baramil Formation of Cretaceous age (Fig. 1). The Nubian sediments are characterized by a wide exposure in the study area and have to form of a half-graben infill controlled by the Timsah fault zone having the NE–SW trend. The sequence dips gently to the SE and imbricate stacking normal faults progressively down steps the sequence such that the upper formations is preserved as sub-parallel elongate strips.

**Figure 2:**
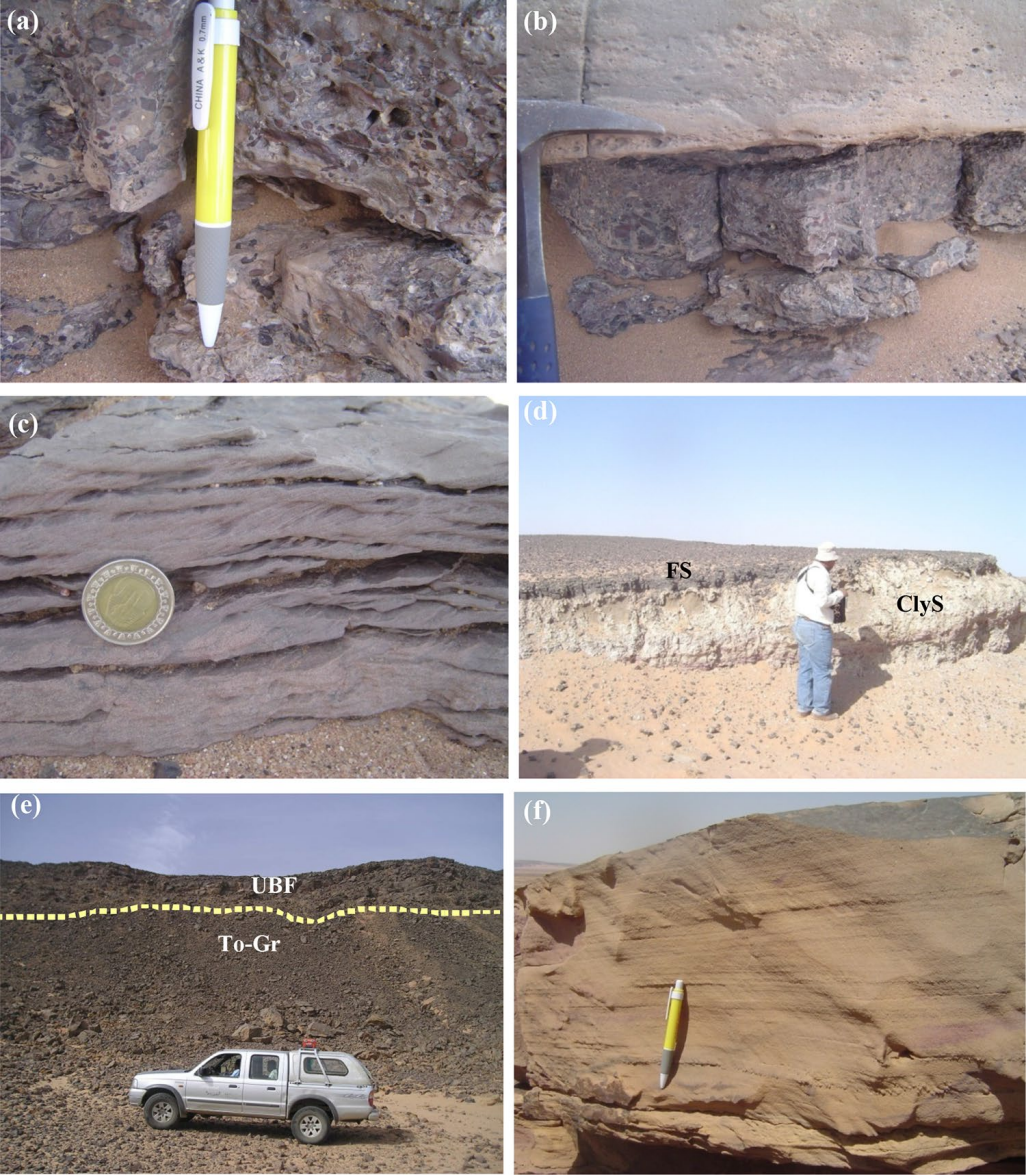
(**a**) The non-oriented and poorly sorted pebbles in conglomerate at Timsah Formation, (**b**) Basal conglomerate underlies graded bedding sandstone, (**c**) Homogeneous erosional cosets of trough cross-stratification, Timsah Formation, (**d**) Clayey sandstone (ClyS) underlies pebbly ferruginous sandstone (FS) at Timsah Formation, (**e**) Non-conformable surface between tonalite-granodiorite (To-Gr) and Um Baramil Formation (UBF), (**f**) Yellowish to gray laminated sandstone of Um Baramil Formation.

**Figure 3:**
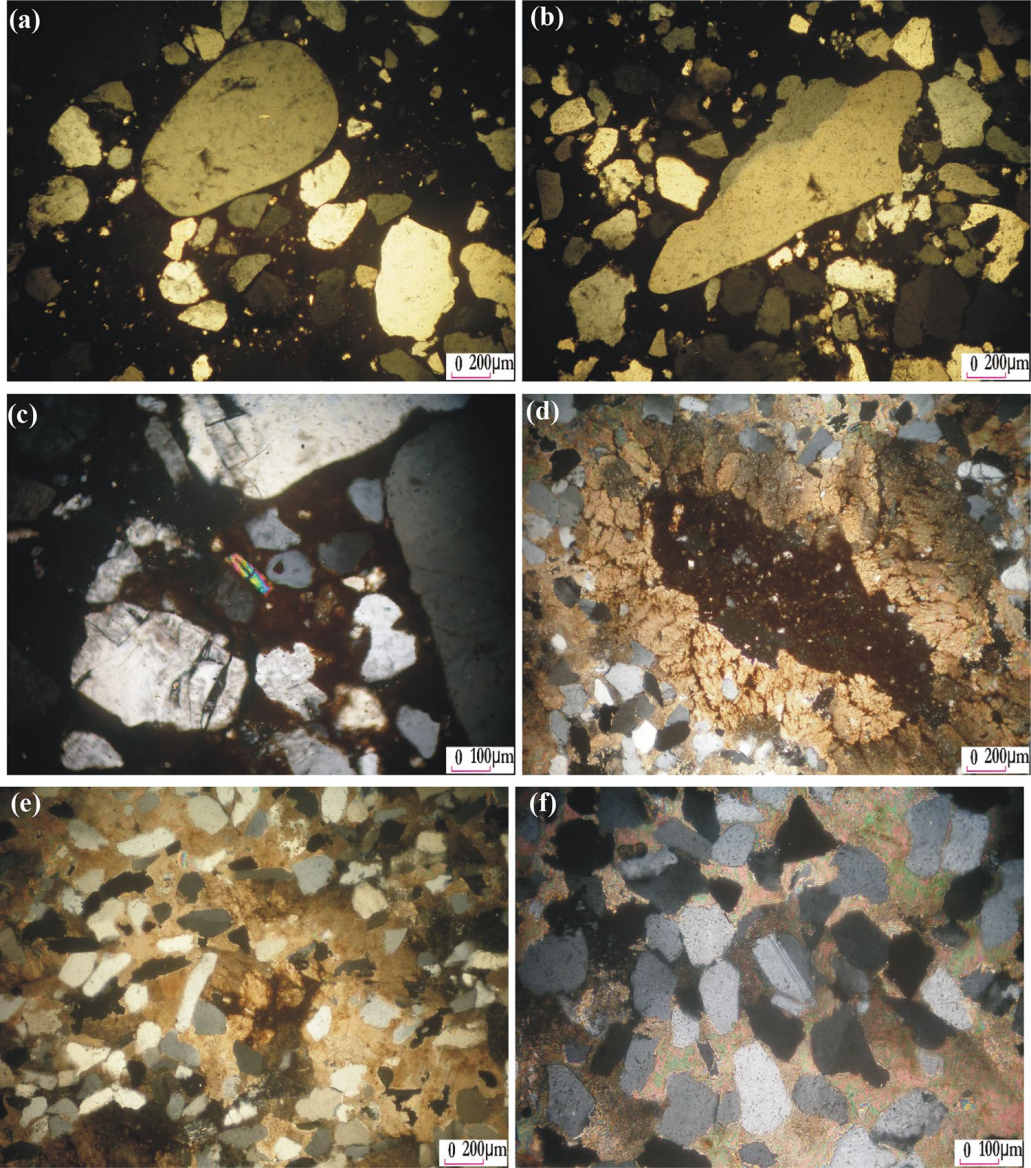
(**a**) Poorly sorted sandstone composed of rounded quartz grains surrounded by subrounded to subangular quartz grains in ferruginous sandstone, (C.N.); (**b**) Angular grains of quartz crystals in ferruginous sandstone, (C.N.); (**c**) Zircon crystal embedded in iron oxide matrix and surrounded by quartz grains in ferruginous sandstone, (C.N.); (**d**) Elongated cavity filled by opaque minerals and surrounded by calcite, greywackes, (C.N.); (**e**, **f**) Greywackes consist of quartz and few plagioclase cemented by carbonates-rich matrix (C.N.).

The Timsah Formation unconformable overlies the Abu Ajjaj Formation and overlains by the Um Barmil Formation^29^. Timsah Formation attains 7.5 m thick, and comprises basal conglomerates, cross-bedding sandstone, pebbly ferruginous sandstone, gradded bedding sandstone and clayey sandstones (Fig. 2a–d). The basal conglomerates are composed of a few bands of gravelly sand or sandy gravels overlying tonalite-granodiorite rocks. The gravels are cemented by white calcareous materials, clayey sandstone or pigmented sand. The pebbles within the conglomeratic bands are mostly composed of quartz characterized by white, yellow, pink or faint brown to reddish brown colors. They vary in shape from semi-equiaxial to triaxial, angular, sub-angular to sub-rounded, range in diameter between 1 and 15 cm and show a concave bottom. The cross-bedding of the most sets range from 30 to 50 cm thick. Traces of laminae are commonly curved and tangential to the underlying erosion surface. The true dip of the cross laminae in their steepest parts commonly range between 15° and 20° to the south. Pebbly ferruginous sandstone bands are dark brown, reddish brown, dark gray and greenish gray colors. They are sub-rounded to rounded, spherical, nodular, rod-shaped and range in diameter from 1 to 3 cm. They are composed of quartz cemented by calcareous and pigmented sands. Gradded bedding sandstone overlies gravelly sandstone beds with the grain size always decreasing upwards. The pebbles show a common orientation and usually lay down with their long axes parallel to the bedding planes. Clayey sandstone is very fine grained, dark gray to grayish green colors. It is associated horizons of iron concentrations range in diameter from 2 to 3 cm as thinly beds. These horizons are composed of dark brown to reddish brown ferruginous sandstone. Clayey sandstone horizon underlies pebbly ferruginous sandstone and cross cut by NNW–SSE strike slip sinstral fault (Fig. 3d). Um Baramil Formation is the most extensive exposure of Nubian sandstone in the investigated area. It overlies Timsah Formation and in other parts overlies the tonalite-granodiorite with non-conformity surface (Fig. 2e). This formation attains 70 m in thickness and comprises three beds (a) yellowish to dark gray sandstone; (b) kaolinitic sandstone and (c) pebbly ferruginous sandstone. The lower part composed of yellowish to dark gray sandstones characterized by coarse grained, massive, laminated, yellow to dark gray colors and their thickness attains 7 m (Fig. 2f). The kaolinitic sandstone horizons attain 2.5 m and characterized by its higher content of radioactivity than the other two beds of yellowish to dark gray and pebbly ferruginated sandstones. Pebbly ferruginated sandstones are fine- to medium-grained, dark brown to pale brown colors. They attain 13.5 m thick. The three horizons are repeated along this formation. Timsah and Um Baramil formations are traversed by N–S strike slip sinstral faults.

## Material and methods

### Ground GS-256 spectrometer

The ground survey gamma-ray spectrometry measurements made over Nubian sandstone rock at Fileita, Southeastern Desert, Egypt. All ground gamma-ray spectrometric measurements were made using a portable Geophysica Brno GS-256 spectrometer. The spectrometer has a 0.35 L sodium iodide (NaI) thallium detector. It was adjusted on 150 s to establish a stable spectrum for radioelement measurements especially for K %, eU ppm and eTh ppm as well as total count (T.C) (Ur, Unit of radioelement concentration). The measured spectrometer data were repeated three times in each site to have a good statistics for the three radioelements. The spectrometer integrates horizontal surface 1 m diameter of the studied sandstones. It is well-calibrated on artificial concrete pads of K, eU and eTh at the Nuclear Materials Authority of Egypt as reported by Grasty et al.^37^.

### Laboratory measurements

To determine the radiological hazard indexes, the natural radioactive content of selected 73 samples was investigated. The radioelement of ^238^U, ^232^Th, and ^40^K were detected. To obtain the secular equilibrium of radionuclides in the ^238^U and ^232^Th series, the samples were sealed to avoid leakage of ^222^Rn and ^220^Rn, and, then, measured after 1 month. A gamma spectrometer with a 76 × 76 mm^2^ NaI(Tl) crystal and a voltage level powered photomultiplier tube (PMT) were used for rapid, non-destructive, and effective radiation detection in the granitic rocks (GR) samples^38^. The pulse management and information analysis apparatus included a multi-channel analyzer and a spectroscopic amplifier, and it was connected to Gamma Vision software for the analysis of the spectra. Gamma energies from the nuclei of the ^238^U, ^232^Th, and ^40^K are 1764 keV, 2614 keV, and 1460 keV respectively. Energy and efficiency calibrations were obtained using three multi-γ-ray sources with known activity and energy values from 59.5 to 1332.5 keV. The range of calibration efficiency is varied from 0.5 to 2%. The granitic samples were measured for 2000s using the minimum detection limits (MDL) of 2, 4, and 12 Bq kg^−1^ for ^238^U, ^232^Th, and ^40^K, respectively. The MDL for ^238^U, ^232^Th, and ^40^K are calculated for each sample by Eq. 1^39^.

1 $$MDL = \frac{2.7 + 4.65 \surd B }{{ M\varepsilon I_{\gamma } t}},$$

where B is the count of the background below the peak of interest, ε is the absolute value Efficiency, Iγ is the intensity of the gamma rays and t is counted. Time (seconds).

Table 1 shows the commonly used radiological hazard indexes estimated in the present study^40^.

**Table 1:** Used radiological hazard indexes for Nubian Sandstone from Fileita, South Eastern Desert, Egypt.

Parameter	Definition	Formula
Absorbed dose rate *D*_*air*_ (nGy h^−1^)	Dose rate exposure in the air at 1 m from radiation sources due to the concentrations (A) of ^238^U, ^232^Th and ^40^K	D_air_ = 0.462 A_U_ + 0.604 A_Th_ + 0.0417 A_K_
Outdoor annual effective dose *AED*_*in*_ (mSv y^−1^)	Monitor of the radiation exposure indoor and outdoor during a stationary period (1 y)	AED_out_ = D_air_ (nGy h^−1^) × 0.2 × 8760 (h/y) × 0.7 (Sv/Gy) × 10^–6^ (mSv/nGy)
Indoor annual effective dose AED_out_ (mSv y^−1^)		AED_in_ = D_air_ (nGy h^−1^) × 0.8 × 8760 (h/y) × 0.7 (Sv/Gy) * 10^–6^ (mSv/nGy)
Excess lifetime cancer risk *ELCR*	Probability of developing cancer over a lifetime at a given exposure level	ELCR = (AED_in_ + AED_out_) × 70 y × 0.05

## Results and discussion

### Petrographic studies of Nubian sandstone

Microscopically, ferruginous sandstones are poorly sorted, fine- to medium-grained. They are composed essentially of quartz grains and cemented by iron-rich fine-grained. Quartz forms subrounded, subangular, to angular grains of various size, (Fig. 3a, b), up to 50% of the rock volume. Zircon form euhedral prismatic crystals distributed in the ferruginous matrix (Fig. 3c).

Greywackes are fine- to medium-grained, poorly sorted and consisting mainly of quartz and feldspar embedded in a fine -grained matrix of quartz, feldspar, sericite and opaques. Quartz occurs as subrounded to subangular crystals embedded in carbonates (calcite)-rich matrix. It forms up to 40% of the rock volume. Some elongated cavities are filled by iron oxides and lined by carbonates (Fig. 3d). Feldspars are represented by plagioclase and microperthite. Plagioclase is fresh to slightly saussuritized and shows lamellar twinning. It is common together with quartz grains in the carbonate-rich matrix (Fig. 3e, f).

### Distribution of the radioelements

#### Nubian sandstone

Timsah formation comprises different rock types including basal conglomerate, cross-bedding, pebbly ferruginous and clayey sandstones (Table 2). Basal conglomerates are characterized by low radioactivity level, which ranges from 5.3 to 7.8 Ur for T.C, 0.2 to 1% for K, 2.2 to 4.8 ppm for eU, 5.9 to 13.3 ppm for eTh. Meanwhile eU-(eTh/3.5) value ranges from ^−^1.4 to 2.54 ppm, and eU/eTh ratio ranges from 0.18 to 0.61. Cross-bedding sandstones show that eU range from 1.8 to 5.1 ppm with a normal distribution of the radioactivity level. It is characterized by low radioactivity level; where K ranges from 0.1 to 0.5%, eTh ranges from 4.5 to 11.7 ppm and eU/eTh ratio from 0.21 to 1.13. Pebbly ferruginous sandstones are characterized by low radioactivity level with 4.4 to 6.9 Ur for T.C, 0.1 to 0.6% for K, 2.4 to 4.4 ppm for eU, and 5.9 to 12 ppm for eTh, while, eU-(eTh/3.5) value ranges from -0.86 to 2.29 ppm. Clayey sandstones are characterized by low radioactivity level with range of radioactivity of 7.4 to 11.1 Ur for T.C, 0.6 to 1.4% for K, 1.8 to 5.5 ppm for eU and 5.8 to 14.9 ppm for eTh. The eU/eTh ratio ranges from 0.19 to 0.4.

**Table 2:** Ranged and mean values of radioelements K, eU, eTh and their ratios of studied rocks using ground GS-256 spectrometer.

	No		T.C Ur	K %	eU ppm	eTh ppm	eU/eTh	eU-(eTh∕3.5) ppm
**Timsah formation**								
Conglomerates	31	Range	5.30–7.80	0.20–1.00	2.20–4.80	5.90–13.30	0.18–0.61	−1.40–2.54
		Mean ± SD	6.91 ± 0.60	0.49 ± 0.18	3.30 ± 0.79	8.98 ± 2.03	0.39 ± 0.15	0.74 ± 1.19
Cross-bedding sandstones	33	Range	4.00–7.30	0.10–0.50	1.80–5.10	4.50–11.70	0.21–1.13	−0.86–3.81
		Mean ± SD	5.00 ± 0.97	0.23 ± 0.13	3.08 ± 0.90	7.42 ± 2.16	0.46 ± 0.24	0.96 ± 1.19
Pebbly ferruginous sandstones	36	Range	4.40–6.90	0.10–0.60	2.40–4.40	5.90–12.00	0.21–0.59	−0.86–2.29
		Mean ± SD	6.13 ± 0.79	0.35 ± 0.17	3.11 ± 0.56	8.69 ± 1.72	0.38 ± 0.12	0.63 ± 0.92
Clayey sandstones	32	Range	7.40–11.10	0.60–1.40	1.80–5.50	5.80–14.90	0.19–0.40	−1.46–1.59
		Mean ± SD	9.81 ± 1.10	0.78 ± 0.19	3.28 ± 1.10	12.54 ± 2.84	0.27 ± 0.07	−0.31 ± 0.95
**Um Baramil formation**								
Yellowish to gray sandstones	37	Range	2.00–4.20	0.10–0.90	0.20–3.40	2.40–6.40	0.03–0.83	−1.63–1.80
		Mean ± SD	3.08 ± 0.69	0.41 ± 0.29	1.58 ± 0.91	4.96 ± 1.16	0.35 ± 0.23	0.16 ± 1.03
Kaolinitic sandstones	31	Range	2.00–7.40	0.10–1.40	0.70–3.40	4.10–6.50	0.11–0.61	−1.10–1.80
		Mean ± SD	4.28 ± 1.67	0.58 ± 0.39	1.95 ± 0.70	5.31 ± 0.83	0.37 ± 0.13	0.43 ± 0.71
Pebbly ferruginous sandstones	35	Range	1.30–3.70	0.10–0.60	0.40–3.30	2.90–6.80	0.07–0.88	−1.34–2.21
		Mean ± SD	2.66 ± 0.74	0.28 ± 0.14	1.71 ± 0.86	4.65 ± 1.22	0.41 ± 0.25	0.39 ± 1.08

Unit of radioelement concentration (Ur).

Um Baramil formation is the upper part of Nubian sandstone comprising yellowish to gray sandstones, kaolinitic sandstones and pebbly ferruginous sandstones (Table 2). Yellowish to gray sandstones are characterized by low radioactivity level. Their range of radioactivity is limited between 2 to 4.2 Ur for T.C, 0.1 to 0.9% for K, 0.2 to 3.4 ppm for eU, 2.4 to 6.4 ppm for eTh. Kaolinitic sandstones have 2 to 7.4 Ur for T.C, 0.1 to 1.4% for K, 0.7 to 3.4 ppm for eU and 4.1 to 6.5 ppm for eTh. The eU/eTh ratio ranges from 0.11 to 0.61, so these rocks don’t show any significance for radioactivity. Pebbly ferruginous sandstones show radioactivity which range from 1.3 to 3.7 Ur for T.C, 0.1 to 0.6% for K, 0.4 to 3.3 ppm for eU, 2.9 to 6.8 ppm for eTh.

#### Radioelements staked profiles of nonconformable surface

**Figure 4:**
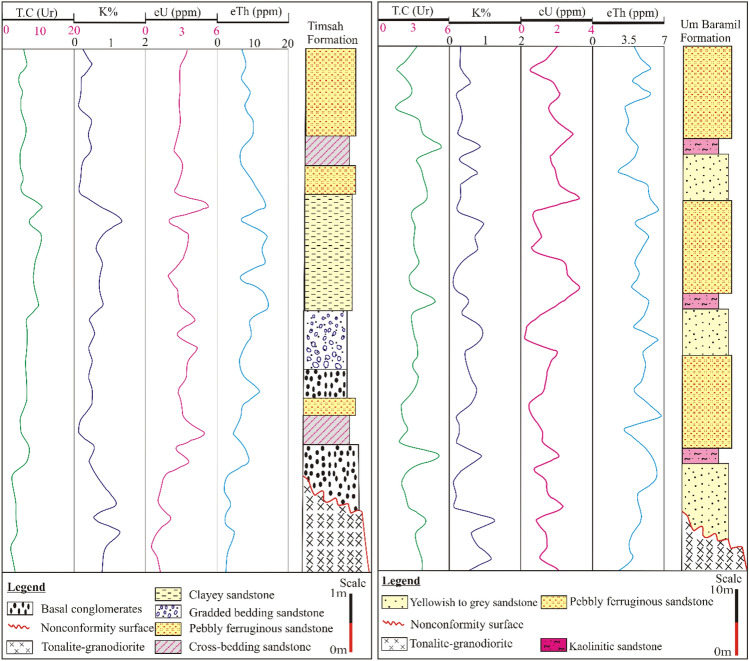
Staked profiles showing radioelements potentiality for Timsah Formation (7 m), and Um Baramil Formation (70 m), along the nonconformable surface between the tonalite-granodiorite and Nubian Sandstone at Fileita area, South Eastern Desert, Egypt.

The two formations are constructing the radioelements potentiality through staked profiles along the nonconformity surface between tonalite-granodiorite and Nubian Sandstone (Fig. 4).

The Timsah Formation overlain granitoids (tonalite-granodiorite) which contain lower range of radioelements distribution; 2 to 4.2 Ur for T.C, 0.5 to 2.2 ppm eU, 1.5 to 4.8 ppm for eTh. Moreover, the nonconformity surface is characterized by low content of radioactivity reached 7.1 Ur for T.C, 1.5 ppm for eU, and 2.6 ppm for eTh. The basal sandstone conglomerates are show higher radioactivity attaining 7.3 Ur for T.C, 3.7 ppm for eU, and 9 ppm for eTh. The lower bed of cross-bedding sandstone shows high peak in the chart attaining 5.1 ppm for eU. The sandstone conglomerates at the base of graded bedding sandstone has high peak reached 4.4 ppm for eU. Clayey sandstone bed shows the higher radioactive zone of the Timsah Formation attaining 11.1 Ur for T.C, 1.4% for K, 5.5 ppm for eU, and 19.9 ppm for eTh. On the other hand, pebbly ferruginous sandstone shows low radioactivity level attaining 6.8 Ur for T.C, 3.5 ppm for eU and 10.4 ppm for eTh.

The lower Um Baramil Formation in some parts has overlain Timsah Formation while it is overlain tonalite-granodiorite in other parts. Tonalite-granodiorite shows low content of their radioactivity reaching 4.1 Ur for T.C, 1.3% for K, 2.1 ppm for eU, and 4.8 ppm for eTh. The nonconformable surface between Um Baramil Formation and tonalite-granodiorite attains 2.6 Ur for T.C, 0.1% for K, 2.4 ppm for eU, and 4.6 ppm for eTh. Yellowish to gray sandstone is characterized by low radioactivity level which reaches 4.2 Ur for T.C, ranges from 0.2 to 3.4 ppm for eU, from 2.4 to 6.4 ppm for eTh. Kaolinitic sandstone shows the highest peak relative to the other beds (Fig. 4) with radioactivity level attaining 5.4 Ur for T.C, 0.9% for K, 2.4 ppm for eU, and 5.8 ppm for eTh. Pebbly ferruginous sandstones are characterized by low radioactivity level of 3.7 Ur for T.C, ranges from 0.4 to 3.5 ppm for eU and from 2.9 to 6.8 ppm for eTh.

Therefore, the nonconformity surface between tonalite-granodiorite and Nubian Sandstone at Timsah and Um Baramil formations does not represent a significant potentiality for their radioactivity.

### Radioactive hazards

Table 3 summaries the results of the content of natural radionuclides sandstone samples. Hereby, the terms eU or eTh denote equivalent of ^238^U or of ^232^Th, meaning the natural radiation emitted by all radionuclides belonging to the decay series of ^238^U (eU = ^226^Ra = ^214^Bi) and ^232^Th (eTh =^228^Th = ^208^Tl), which occur in the analyzed samples^41^. This fact is performed assuming the secular equilibrium of radionuclides, obtained by sealing the samples to avoid leakage of ^222^Rn and ^220^Rn, and, then, measuring the samples after 1 month. In particular, eU ranged from 1 to 15 ppm, eTh ranged from 1.3 to 50 ppm, ^40^K ranged from 0.2 to 5% with means of 5.7 ppm, 14.4 ppm and 3.2%, respectively. The following conversions can be used to obtain the radionuclide content into activity concentration^42^: 1% of ^40^K in rock corresponds to 313 Bq kg^−1^; 1 ppm eU in rock is 12.35 Bq kg^−1^ of ^238^U; 1 ppm of eTh in rock is 4.06 Bq kg^−1^ of ^232^Th.

**Table 3:** Statistics of radiometric results on 73 samples sandstones from Feleita area.

	Minimum	Maximum	Mean ± SD*	CV (%)
eU (ppm)	0.4	5.5	2.6 ± 1.1	40
eTh (ppm)	2.9	14.9	7.3 ± 3.0	41
^40^K (%)	0.1	1.4	0.4 ± 0.3	65
^238^U (Bq kg^−1^)	4.9	67.9	32.0 ± 13.0	40
^232^Th (Bq kg^−1^)	11.8	60.5	29.6 ± 12.2	41
^40^K (Bq kg^−1^)	31.3	438.2	132.6 ± 86.4	65

*SD standard deviation.

**Figure 5:**
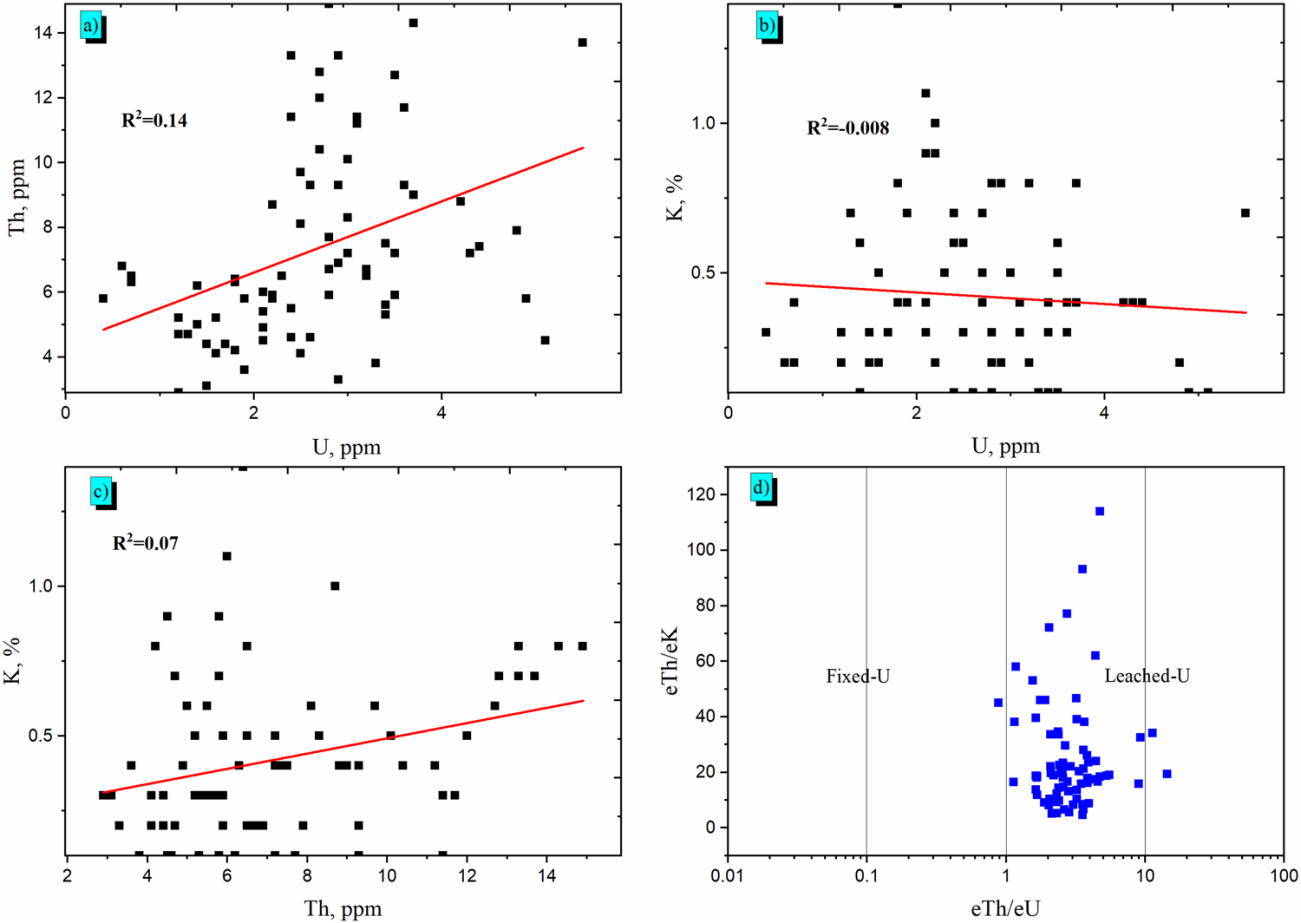
Radiometric analysis in 73 samples of sandstones in the studied region: correlations between (**a**) eTh and eU, (**b**) eK and eU, (**c**) eK and eTh, (**d**) eTh/eK and eTh/eU.

The obtained results in Table 3 show low values in accordance with the literature of sandstones: 3–6 ppm for eU and 8–23 ppm for eTh^43,44^. According to the work^45^, Table 3 also reported the coefficient of variability (CV) that determines how statistically significant are the data from gamma-ray spectrometry. According to the equation from^46^, i.e. CV % = (σ/X) × 100 with σ the standard deviation and X the arithmetic mean, the data tend to have a normal distribution in the study region if the CV is less than 100%. In the Table 3, CV determines the degree of distributions homogeneity of the several radioelement for the sandstone rocks. Table 3 shows that ^40^K distributions are high homogeneous compared to eU and eTh that indicate moderate levels of homogeneity. These values can be explained by the presence of different minerals during geological formations in sandstone samples. Figure 5a illustrates the relationships between eTh–eU, showing a very strong relationship (R^2^ = 0.77). This aspect suggests that uranium and thorium mineralization are controlled by magmatic processes. Figure 5a illustrates the weak correlation between eU and eTh (R^2^ = 0.14). Figure 5b, c show also the weak relation of ^40^K with eU and eTh, respectively. This may be attributed to the alteration processes of feldspars. The relationship between the radioelements exhibits that their distribution are not only magmatic but also hydrothermal alteration. Moreover, in Fig. 5d the eTh/K versus eTh/eU shows that the majority of the samples under evaluation are located in the leached-U sector, indicating that using them as building material or decorations could be safely^47^.

**Figure 6:**
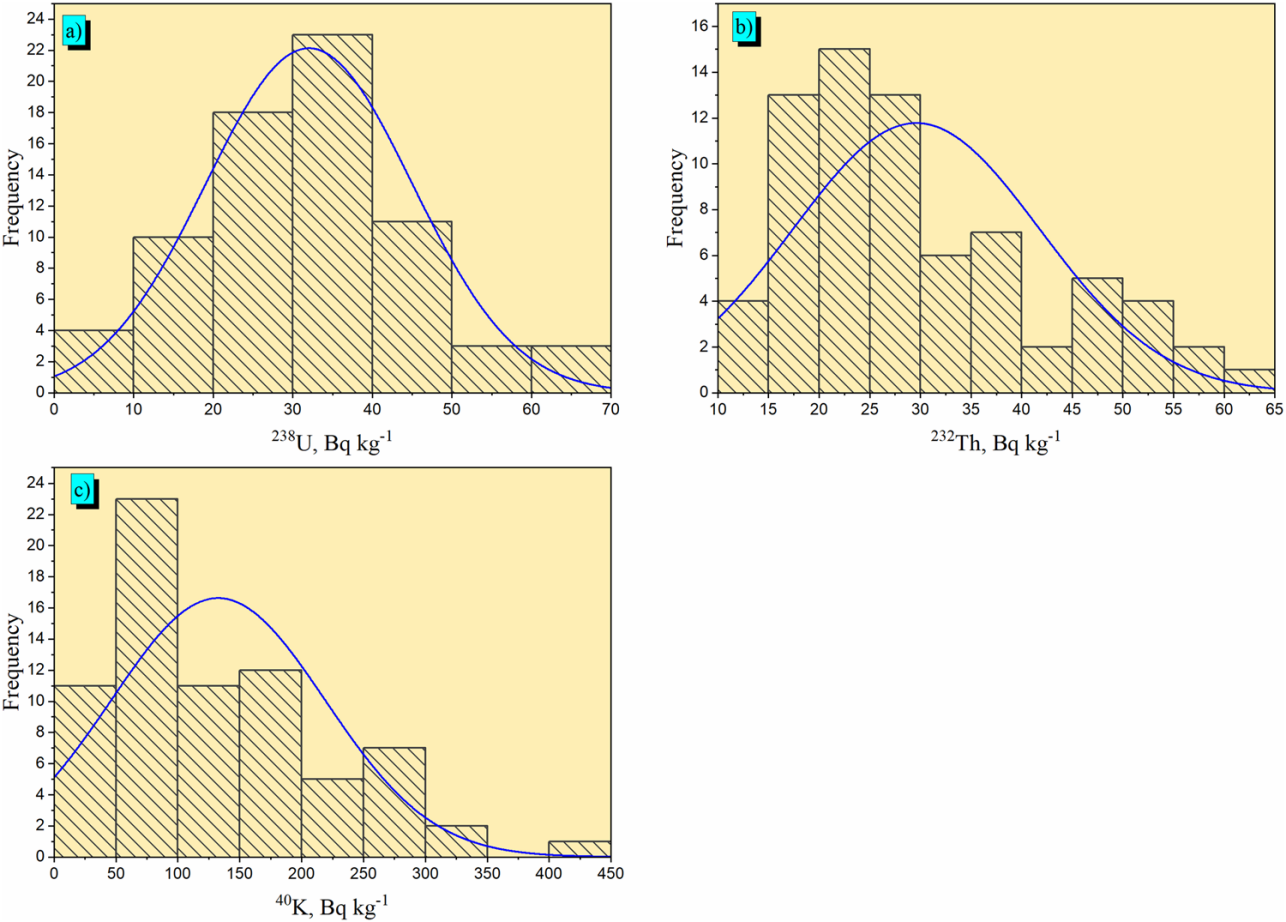
Radiometric analysis in 73 samples of sandstone in the studied region: frequency distribution of the activity concentration (Bq kg^−1^) of (**a**) ^238^U, (**b**) ^232^Th, (**c**) ^40^K.

In the examined sandstone, the radionuclide activity concentrations of ^238^U, ^232^Th and ^40^K in Bq kg^−1^ are also displayed in Table 3. The range of ^238^U, ^232^Th and ^40^K activity concentrations are 4.9–67.9 Bq kg^−1^, 11.8–60.5 Bq kg^−1^ and 31.3–438.2 Bq kg^−1^, respectively. The mean values with standard deviation of ^238^U, ^232^Th and ^40^K activity concentrations are 32 ± 13 Bq kg^−1^, 29.6 ± 12.2 Bq kg^−1^ and 132.6 ± 86.4 Bq kg^−1^, respectively. The obtained results show that the mean data for ^238^U, ^232^Th and ^40^K are lower than the worldwide mean data, which are 33, 45, and 412 Bq kg^−1^, respectively^6,48^. The high radioactive levels of some investigated samples are as a result of weathering, leaching, modification, and alteration processes. A buildup of radioactive minerals, such as staurolite, zircon, rutile, magnetite, ilmenite and garnet are associated with sandstone beds at Fileita according to their dominance^29^. The skewness data revealed an uneven distribution that aligned with the fundamental statistical analysis of radioelement activity concentrations. Favorable findings indicated an asymmetric distribution. Despite referring to their negative conclusions as negative data, the tail of the asymmetric distribution is extended. As a result, the data on ^238^U (0.36) and ^232^Th (0.86) activity concentrations' are positive skewness suggest that the asymmetry is positive. Secondly, the kurtosis values indicate the peakedness of the distribution probability. The activity and normal distributions are asymmetrical, as evidenced by the radioactive materials ^40^K (1.14), which has kurtosis coefficients greater than 1. Conversely, the kurtosis coefficient for sample ^238^U (0.37) and ^232^Th (–0.12) was less than 1, suggesting that the probability distribution is flat. The frequency distribution of ^238^U, ^232^Th and ^40^K in the 73 samples are presented in Fig. 6.

### Radiological hazard indices

The radiological hazard indexes determine the assessment of sandstone from Feleita used as construction materials: in Table 4 are reported the results. The absorbed dose rate D_air_ varied among 18 and 74 nGy h^−1^, with a mean value of 38 nGy h^−1^ that is lower than the UNSCEAR population-weighted average of 59 nGy h^−1^^6^. Consequently, the exposure from these sandstones does not give a considerable radiation dose to the public. Indoor and outdoor annual effective dose AED_in_ and AED_out_ mean values are 0.05and 0.19 mSv y^−1^, respectively, which are lower than the allowable means of 0.40 mSv y^−1^ (indoor) and 0.07 mSv y^−1^ (outdoor)^6^. This means that this long-term low-level exposure is not expected to have negative health repercussions^49^. The excess lifetime cancer risk (ELCR)_,_ i.e. the probability of developing cancer over a lifetime at a given exposure level, is found over the worldwide mean limit of ranges from 0.08 to 0.32 with a mean value of 0.16, where it is significantly lower than the reference limit of 0.0029^40^.

**Table 4:** Radiological hazard indexes in the 73 sandstone samples from Feleita area.

	D_air_ (nGy h^−1^)	AED_out_ (mSv y^−1^)	AED_in_ (mSv y^−1^)	ELCR × 10^–3^
Mean	38	0.05	0.19	0.16
Sd	12	0.02	0.06	0.05
Minimum	18	0.02	0.09	0.08
Maximum	74	0.09	0.36	0.32

### Pearson correlation, principal component and hierarchical cluster analysis

Correlations between ^238^U, ^232^Th and ^40^K activity concentrations and radiological hazard indicators in the 73 sandstone samples from Feleita area are studied by using the Pearson correlation analysis (PC) (Table 5). The PC analysis classified the correlations into four categories: weak (0.3–0.49), moderate (0.5–0.69), high (0.7–0.9), and extremely strong (> 0.9) ^43^. Weak correlations are between ^238^U with ^40^K (R^2^ = 0.-0.07) and ^232^Th (R^2^ = 0.40). The correlation between ^40^K and ^232^Th is weak (0.28). These low correlations are explained by the investigation of staurolite, zircon, rutile, magnetite, ilmenite and garnet are associated with sandstone beds at Fileita according to their dominance^29^. Table 5 shows that there are high connections between ^238^U and ^232^Th with the radiological hazard indexes (≥ 80%). As a result, the main causes of the negative health effects associated with the gamma radiation which produced from the high activity concentrations of uranium and thorium of some sandstone samples.

**Table 5:** Pearson correlation among radionuclides content and radiological hazard indexes in the 73 samples of sandstone from Feleita region of Egypt.

	^238^U	^232^Th	^40^K	D_air_	AED_out_	AED_in_	ELCR
^238^U	1	0.40	−0.07	0.71	0.71	0.71	0.71
^232^Th	\\	1	0.28	0.88	0.88	0.88	0.88
^40^K	\\	\\	1	0.42	0.42	0.42	0.42

**Figure 7:**
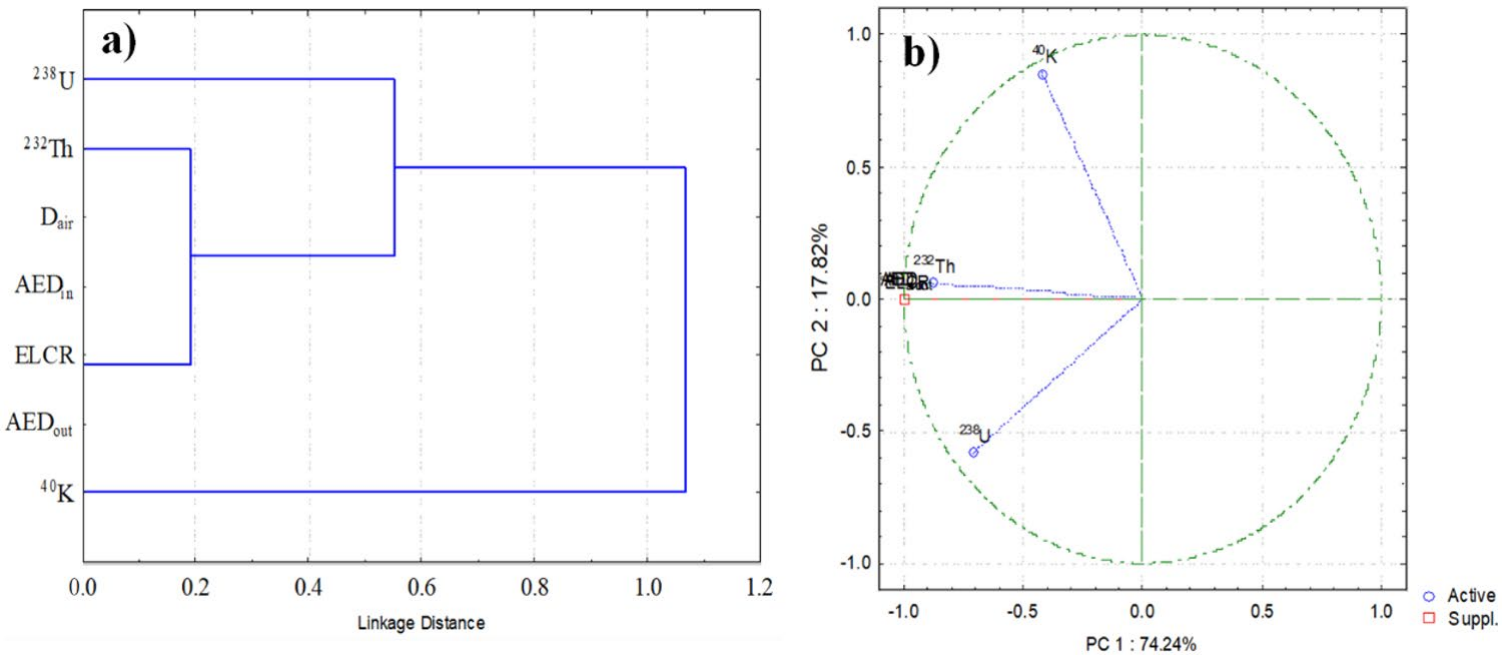
Graphical representation of PCA analysis (**a**) and dendrogram from hierarchical cluster analysis (**b**) of radionuclides content and radiological hazard indexes in 73 samples of sandstone from Feleita area.

The principal component analysis (PCA) is also performed to further analyze the relation among the ^238^U, ^232^Th and ^40^K activity concentrations and radiological hazard indicators in the 73 samples of sandstone from Feleita area^50^. Component 1 (PC1) and component 2 (PC2) are obtained and shown in Fig. 7. From the figure is clear that PC1 is heavily loaded with the activity concentrations of ^238^U and ^232^Th in relation to all radioactive components (74.24%). In fact, in the sandstones at the work location, ^238^U and ^232^Th were the primary naturally occurring radioactive contributions. Contrarily, the PC2 load for ^40^K (17.82%). This is also evident in the radiological hazard indexes behavior^43,51^. The hierarchical cluster analysis (HCA) is a data categorization system that use multivariate algorithms to distinguish between several types of data. The Euclidean distance between radiological hazard indexes and radioactive activity concentrations is performed^52^. The HCA output is the dendrogram of the investigated data in Fig. 7, obtaining three major clusters. ^238^U is present in cluster I, ^232^Th is present in cluster II, along with radiological hazard indexes, and ^40^K is present in cluster III. According to HCA, the principal gamma radiation sources (^238^U and ^232^Th) in the sandstone samples of the analyzed area are connected with the radiological danger factors, which can be explained by this^47^.

## Conclusion

This study examines the distribution and potential mechanism of occurrence for the radioactive substances in sandstone at Feleita area, Egypt. A range of radiological hazard parameters were estimated, such as the dose rate, the annual effective dose and excess life time cancer risk. The study of the correlation between radionuclides and their corresponding radiological hazard variables involved the use of multivariate statistical techniques like Pearson correlation, principal component analysis, and hierarchical cluster analysis. These findings indicated the activity levels of ^238^U, ^232^Th and ^40^K in the sandstones are 32 ± 13, 29.6 ± 12.2, and 132.6 ± 86.4 Bq kg^−1^, respectively. They are lower than the global limit 33, 45 and 412 Bk kg^−1^. Sandstones’ radioactivity is primarily caused by the uranium, thorium and potassium. Building materials made from sandstones are not likely to present any danger for the public.

## Data Availability

All data generated or analyzed during this study are included in this published article.
